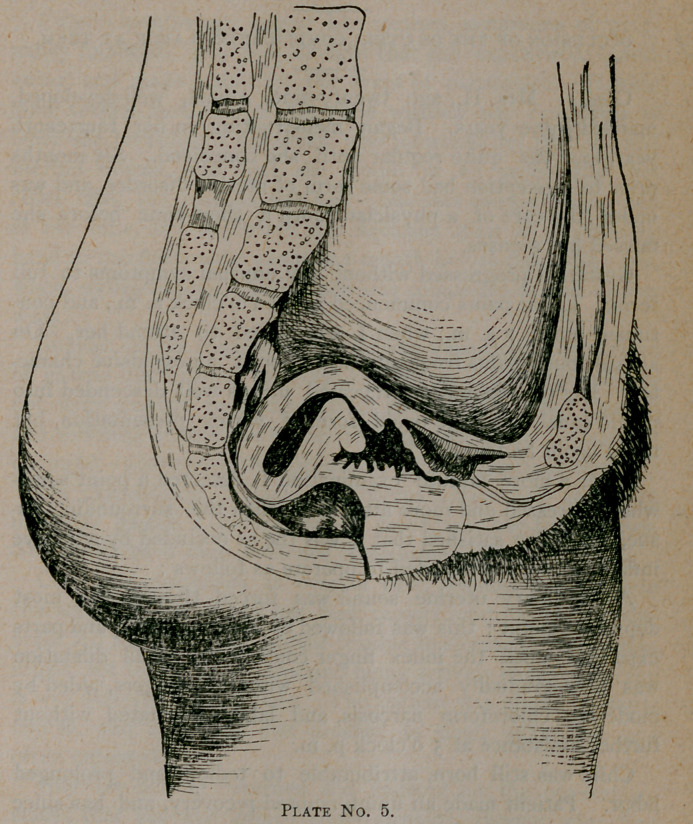# Ante and Retro-Positions of the Uterus—Their Pathology, Symtomatology and Treatment

**Published:** 1893-08

**Authors:** W. W. Stewart

**Affiliations:** Columbus, Ga.


### Southern Medical Record.

A MONTHLY JOURNAL OF MEDICINE AND SURGERY.

VOL. XXIII. ATLANTA, GA., AUGUST, 1893. No. 8.

### Original Articles.

### ANTE AND RETRO-POSITIONS OF THE UTERUS—THEIR PATHOLOGY, SYMTOMATOLOGY AND TREATMFNT.

BY W. W. STEWART, M. D., COLUMBUS, GA.

To understand fully the malpositions of this organ we must first make ourselves familiar with its normal position, and second, with its supports.

The position as seen in this plate, No. 12 (bladder and rectum empty), represents the normal position both in the virgin and the parous woman. In the virgin it is more anteflected, the normal position as described by Schultz when a woman is standing upright her bladder and rectum empty, her uterus is nearly horizontal; is more or less anteflected and turned a little to the right.

This position is modified to a certain extent by the repletion of bladder and rectum.

The next question in order is what holds the uterus to its normal axis. This question is plainly answered in plate No. 1. Here we have a diagramatic outline of all but two of its supports. The two not represented here are the vaginal walls and gravitation with intra-abdominal pressure.

Thus we see the uterus is an organ nicely balanced on elastic cords well cushioned above by the abdominal viscera and below by the vagina, which also is supportive.

As to the etiology of displacements, I can think of no bet-

PLATE No. 12.

ter classification than that of Thomas and Munde, which I give below as taken from their book:

The general cause of uterine displacements may thus be tabulated:

1. Any influence which increases the weight of the uterus.

Influences increasing weight are congestion, tumors in walls or cavity, pregnancy, excessive growth of any of the component parts, subinvolution.

PLATE NO. 1.

2. Any influence which weakens the uterine support. These are rupture of perineum and posterior vaginal wall, weakening of vaginal walls from subinvolution and over distention, stretching of uterine ligaments, relaxation of pelvic fascia, abnormally large pelvis, any influence impairing the sustaining power of the abdominal walls.

3. Any influence which displaces the uterus by pressure; as tight clothing, heavy clothing supported on the abdomen, muscular efforts, abdominal tumors, pelvic inflammatory exudations, repletion of bladder and rectum.

4. Any influence which displaces the uterus by traction ; as contracting adhesions following pelvic imflammations, either cellular or intraperitoneal, cicatrices in vaginal walls, shortening of uterine ligaments, natural shortness of vagina and uterine ligaments, prolapse of vagina, rectum or bladder.

It is an interesting fact to note that the cervical and cervico-corporal flexions are most frequent in the nulliparous woman, while the corporal form is most often found in the multiparous.

Rotroflexions are most usually found in cases in which there has been a weakening of the tone of the uterine wall, although it is often caused by direct force whether of rapid or slow development. The uterus may be either anteflexed, retroflexed or lateroflexed, or ante-lateroflexed, or retro-lateroflexed. All of these varieties have various complications, the most prominent of which is ascent and descent.

There are still two other forms, ante-position with retroflexion and retro-position with anteflexion.

Symptoms : Pain in back and loins, weighty, dragging sensation in the pelvis. Constipation in retro-position—vesical and rectal tenesmus in both. Great fatigue from walking, much complained of below the knees, lassitude and inability to lift weights, leucorrhœa and other signs of congestion.

In addition to these I must mention a uterine symptom which is quite interesting, and at the same time of great aid often in obscure cases where sufficient direct symptoms are not complained of to suggest to one, not accustomed to handle many of these cases, the advisability of an examination.

1. Pain, of a bearing down nature in the top of the head, caused by endometritis or vesical irritation.

2. Pseudo-angina with palpitation of gastric origin.

3. Irritative or indolent digestion with dilitation of the stomach. I would beg of you to remember this fact, for it will, I am sure, prove not only of interest but of profit to all. This dyspepsia is caused by the endometritis associated with flexion. The explanation of this is found in the peculiar richness of the sympathetic innervation of both the uterus and stomach, and is of reflex origin. Its line of symptoms are nausea, loss of appetite, vomiting directly after eating, it being rather regurgitant in character ; flatulency, which occurs in the form of a chronic tympanites, the patient's stomach continuing to enlarge though she may have lost flesh.

4. A dry, hacking cough, which is paroxysmal and comes usually in three or five coughing efforts at a time. So troublesome is this, that the patients, who suffer therefrom, cannot sleep at times. No inflammation of chest, pharynx or larynx can be detected, and it disappears with the uterine trouble.

5. Pain in either breast, usually the left.

6. Pain of a dull character in the wrists, inner side of the palms, and last phalanges.

7. Hysterical phenomena whose name is legion.

8. The facies uterina, which you will recognize in a habitual chloro-anemic color, muddy complexion, dark circles under the eyes, together with a pinched suffering face, which completes the picture. In addition to these there are, of course, many symptoms peculiar to each individual case.

To illustrate this fully I will mention a case of mine which bears upon this point: Miss———, age thirty; occupation dressmaker, came under my care for dyspepsia and vesical tenesmus. For five years had been a great sufferer with pelvic pains, vesical and rectal tenesmus. Headaches, occipito-parietal and parietal in location; constipation at times, at others long continued attacks of diarrhœa, spitting up of food directly after eating, tympanites so marked that she was ashamed to go on the street. Had lost flesh for months prior to coming under my care. Measurement of stomach at umbilicus twenty-nine; three inches below, thirty-five inches. Muscular walls tense. Leucorrhœa was quite bad causing great discomfort and decided excoriation. Urine alkaline, no albumin or sugar. Stomach would easily hold six glasses of water without discomfort, when loud succussion sounds could be elicited. Percussion showed the stomach greatly dilated. Abdomen generally hyperæsthetic. Uterus anteflexed with decided endometritis. Patient was taken off of all starchy and saccharine food and given proper tonics and uterine trouble properly treated. When discharged had gained fourteen pounds, and stomach measurements were as follows: At umbilicus twenty-two; three inches below, twenty-seven inches. Stomach was still slightly dilated, but caused no dyspeptic symptoms. All other symptoms disappeared.

PATHOLOGY.

Flexions and versions, either anterior or posterior, are often congenital, caused by an excessive nutrition in the anterior or posterior wall, as the case may be ; the excessive growth causing a convexity in the side most highly nourished, and a concavity in the opposite.

Rokitanskey has proven that in a perfectly developed uterus, weakening in its walls is often caused by endometritis, which creates an inward growth of the utricular glands into the submucosa near the os internum, which in consequence undergoes atrophy and enfeeblement.

Klobe gives as a frequent cause cystic degeneration of the cervical glands, which from their increased size and subsequent pressure, bursting thereby, cause a collapse of tissue in the formerly dense framework of the uterus, leaving in its place a flaccid net-like areola tissue incapable of sustaining the uterus in its normal position.

The uterus being once flexed, either in the anterior or posterior position, there at once arises an acute congestion followed by subacute and chronic in the majority of cases. Especially is this true in cases of sudden displacement, and it holds good to a modified degree in all cases.

This is caused by veins with their flaccid walls being closed at the point of flexion, while the arterial walls, being rigid, remain open and continue to convey blood into an organ already full, thus causing the acute congestion. The flexion cuts off the return flow through the hypogastrics, thereby throwing all the work upon the inadequate spermatic veins. As a result of this the pampiniform flexus is constantly over-distended, and an œdematous boggy condition is set up in ovaries, tubes, broad ligaments and uterus, making the flexion worse, at the same time setting up a tendency to hydrosalpinx and cystic degeneration and formation in ovaries and broad ligaments.

One of the most prominent pathological lesions with which we have to deal as causative in displacements is slight infection at parturition, causing subinvolution with metritis and parametritis, followed by contraction of uterine ligaments.

After thus hurriedly considering the etiology, pathology and symptomatology of these troubles, we will now take up the cases themselves and discuss them, endeavoring at the same time to bring out the treatment in each case.

Here in plate No. 2 we have a simple retroversion with beginning prolapse caused by subinvolution and relaxation of the uterine ligaments, vaginal walls and perineal fasciae. This is a case in which, if of recent origin, we can look for good and quick results. Having made first a clear diagnosis of the cause (and I would state here that is the keynote to the successful treatment), we will begin our treatment, which, in all displacements, will be along certain general lines. These we will give now and not repeat :

1. Remove all weight from the hips and constrictions from the waist by using skirt supporters.

2. Measure your patient at the line of the umbilicus, or just above, and secure for her a corset waist two iches smaller than waist measure.

3. If abdominal walls are pendulous and relaxed use an abdominal belt made for the patient in question.

4. Require a regular amount of daily exercise, to be gradually increased, that will not overtax the strength.

5. Order alternative hot and cold douches to be taken in the

PLATE NO. 2.

dorsal position. Use the fountain syringe only, with one quart in quantity—patient to remain in recumbent position for thirty minutes after taking.

The alternate hot and cold douche tends to cause contraction of the muscular fibres of the uterus and uterine ligaments, thus bringing about good nutrition and muscular tone, as is produced by electricity. The cold douches should be taken last.

6. Guard well and build up your patient's general condition.

As to the special treatment of this case in hand, the patient should be placed in the genu-pectoral position and uterus replaced. This position should be well explained and taught the patient, with instructions to assume it three or four times daily, and at the same time to open the vagina and allow it to distend with air, then to take successive long breaths and remain in this position for ten minutes, then gradually lay over on her side and remain quiet for a while, thus a replacement three or four times daily is accomplished.

If the uterus or pelvic organs are very tender the organ should be supported with a cotton wool tampon made like this one I show you, and soaked in glycerine and rose water aa one-fourth Lloyd's hydrastis one-half, placed directly behind the cervix; transversely two or three smaller plegets are placed in front; these to remain for not over forty-eight hours, when it should be replaced by fresh ones.

If patient can tolerate it a hard rubber Hodge pessary is better, as it allows the douches to be taken without its removal. This pessary should be *fitted* to the patient as follows: Measure, while in knee-chest position, distance from posterior wall of cul-de-sac of Douglas to inner surface of pubic arch ; then the transverse width. This can be nicely done with dressing forceps. Having these measurements, select the pessary you wish and emerse it in boiling water till soft; then mould to suit the case by measurements taken. It should not give pain, and should scarcely be felt.

Now, this patient is suffering from subinvolution ; so we will give her, in addition to a good tonic, fifteen to twenty drops of Squibb's fluid extract of ergot t. i. d. and ten grains of potassium bromide in solution, the latter to be given after meals and to be continued till uterus is normal in size.

If endometritis exists, begin all treatment by a good curetting, and pack uterus with a long strip of iodoform gauze, to be removed on the third day.

The laws of antisepsis should be carefully carried out throughout the treatment.

PLATE No. 3.

Now in this plate, No. 3, we have a very interesting case, bringing out strongly another frequent cause for displacement.

Miss G——, aged twenty-five, when twelve years old fell upon a steelyard hook, the hook entering the vagina and tearing the tissues badly, its course being upward and outward against the inner surface of the pubic bone.

Had been a sufferer in the long line of symptoms so familiar to us all, till she fell into my hands five months ago. On examination, the condition you see here represented was found. The vesico-uterine ligaments were taut; cervix fixed in its anterior and prolapsed position; uterus retroverted.

Patient placed in genu-pecotral position and uterus returned as far as practical to its normal position. Then I began to stretch the vesico-uterine ligaments by daily pushing the cer-

FIGURE OF 8 PESSARY.

vix backward as far as she could tolerate, and after ten days of this treatment succeeded in getting it back sufficiently to use a figure of 8 pessary, which she wore with comfort for one month, when length was increased till uterus was in normal position and freely movable.

The uterus was decidedly sinistro rotated, and when pessary was removed would immediately return to its former position to a certain extent.

Thinking it was one of those cases in which Alexandre's operation would be of benefit, I operated and found that the hook, which entered the vagina on the right side, had torn out the round ligament, causing the sinistro displacement; so was

FIGURE 8 PESSARY IN POSITION.

compelled to sew up the wound and depend upon a figure of 8 pessary to do the work.

This same displacement with similar etiology we find in women who have passed through protracted labor when the head has pressed against the pubic arch, setting up a cellular inflammation, which is followed by contraction of the vesico-uterine ligaments. In such cases the only method of relief is found in the figure of 8 pessary which should be made by measurements and the length gradually increased till the cervix holds its normal position. No other pessary will suit or benefit such a case. The same contractions I have also found after a prolonged and active cystitis.

PLATE No. 4.

Our next case, gentlemen, represents a complication which is apt to prove interesting.

This represents a case, Mrs. G., age twenty-seven, one child, sent us from Jacksonville, Florida. A case of retroversion with prolapsed tube and ovary and elongated cervix.

This case was one of peculiar interest, as it demonstrated to my mind what persistent care and strict attention to directions would do.

The etiology in this case was general relaxation with subinvolution.

The same general line of treatment was carried out as in case No. 2.

The genu pectoral position was assumed four or five times daily, and cotton wool tampons placed directly behind the cervix once in twenty-four hours. This was kept up for three months; at the end of which time the ovary resumed its normal position and a pessary could be used, which she wore for two months more. The pessary was then removed, the uterus remaining in its normal position.

Case 5. Mrs. P., age thirty, four children, was sent to me from Florida, for bladder trouble and epileptic fits. At birth of last child, three years ago, had considerable trouble, the nature of which could not be learned. Was in labor forty-eight hours when an instrumental delivery was accomplished, after which she had fever for two weeks and was in bed forty-five days. On resuming her duties was troubled with a constant desire to pass water. Pain in loins; bad and almost constant headache, occipito-parietal. Constant leucorrhœa, constipation to a fearful extent.

Two months after resuming her duties, patient was seized with what was diagnosed to be epilepsy of the grand mal type, for which she received constant treatment to no avail.

Attacks grew more frequent and more prolonged. After an attack, would remain unconscious for six or seven hours.

This was her condition augmented by irritated dyspepsia and dilated stomach when I first saw her.

The general condition you see depicted in this drawing.

Uterus retroflexed and drawn backward and upward by sacro-uterine ligaments. Marked endometritis. Roof of bladder taut and paralyzed. Marked cystitis, 20 per cent. by measure of pus in urine.

Perineum torn to second degree which is not shown in this drawing. Cervix bilaterally lacerated. Facies uterina marked.

She was first curetted and uterus packed with iodoform gauze.

PLATE NO. 5.

Six days thereafter cervix was operated upon and in fourteen days perineorraphy was performed. After this had no more epileptic seizures and returned home in good health, the uterus returning to normal position after curetting and other operations mentioned.

(*Continued.*)

## Figures and Tables

**Plate No. 12. f1:**
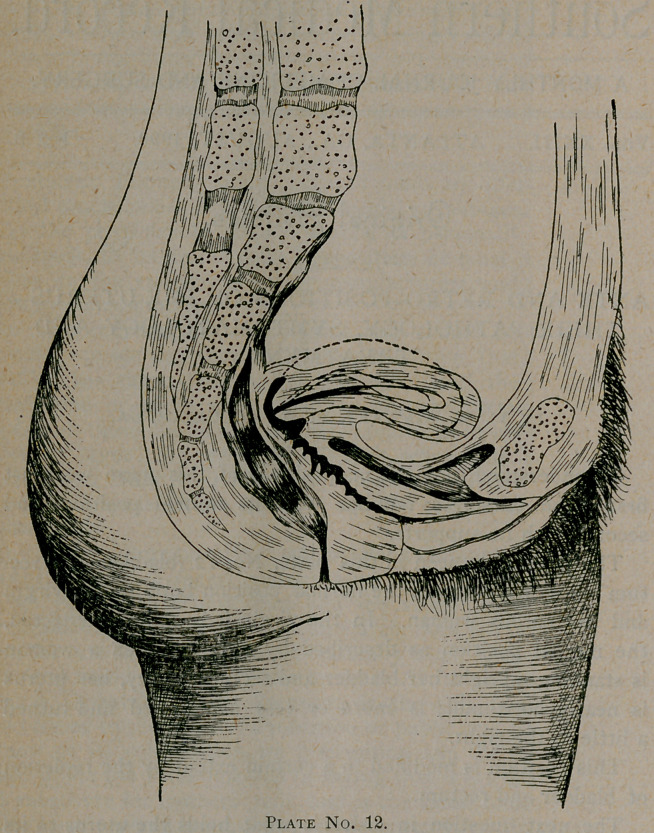


**Plate No. 1. f2:**
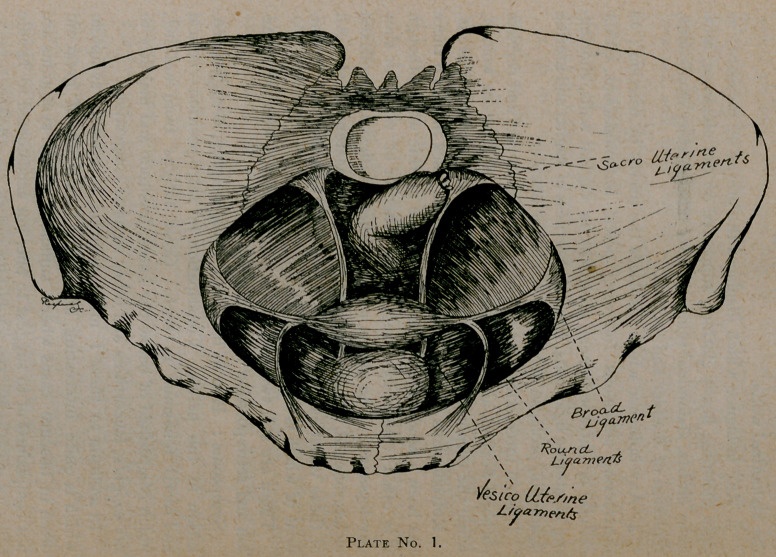


**Plate No. 2. f3:**
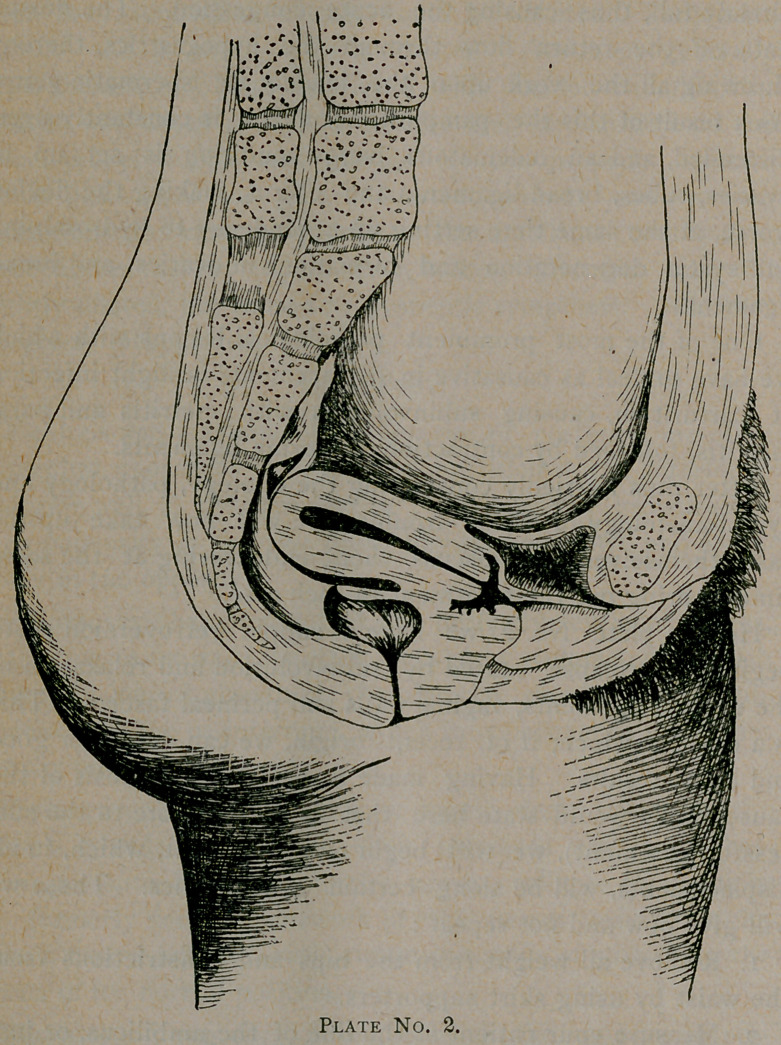


**Plate No. 3. f4:**
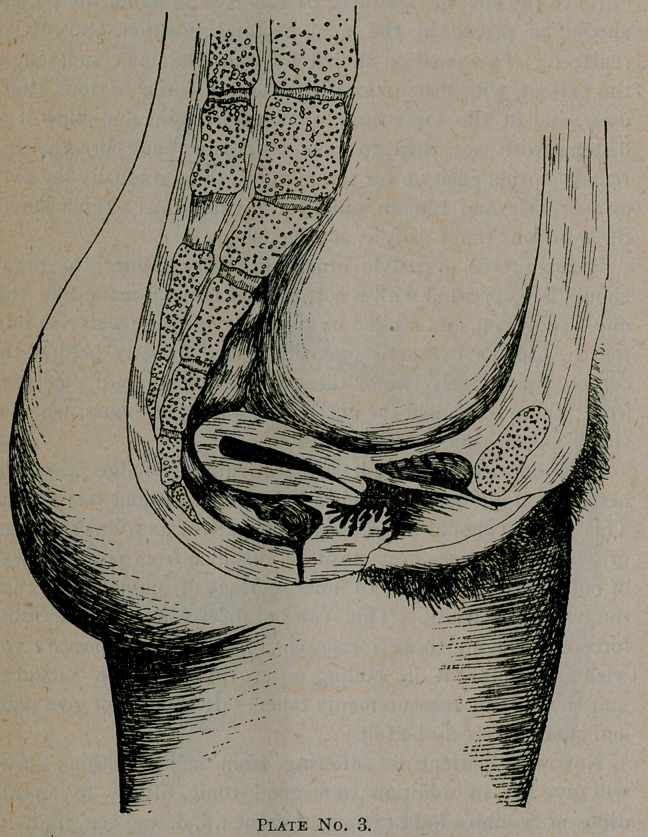


**Figure f5:**
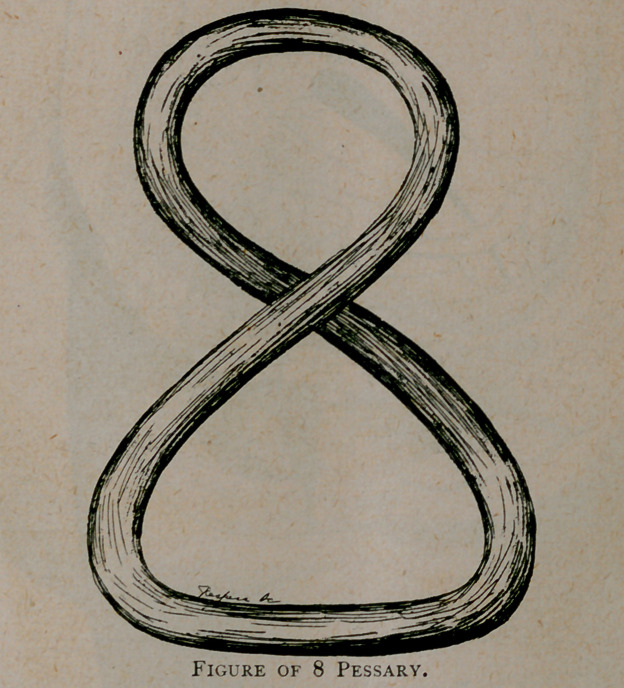


**Figure f6:**
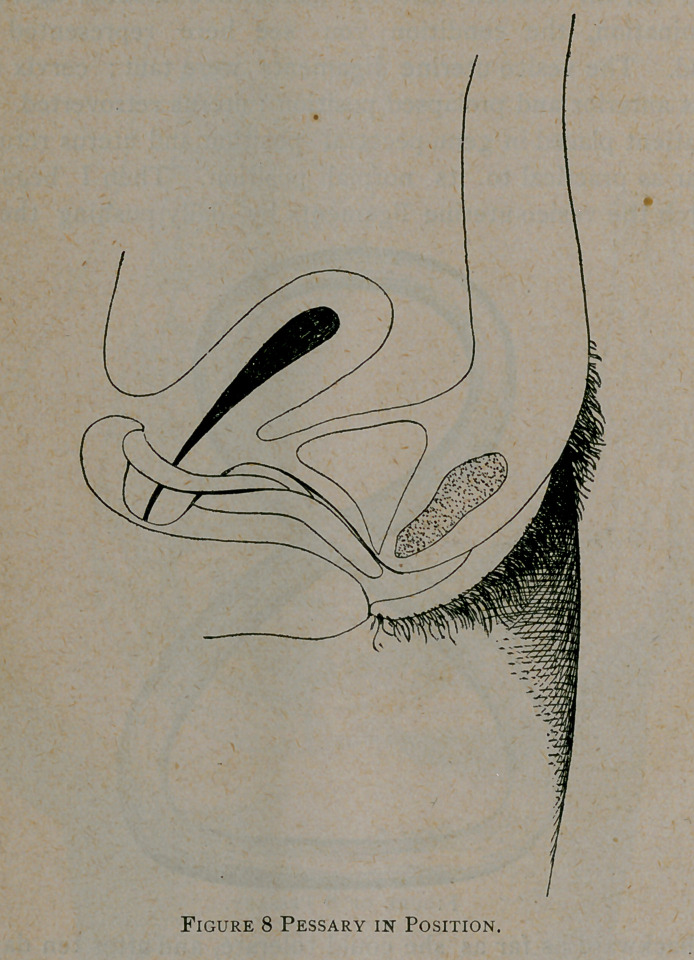


**Plate No. 4. f7:**
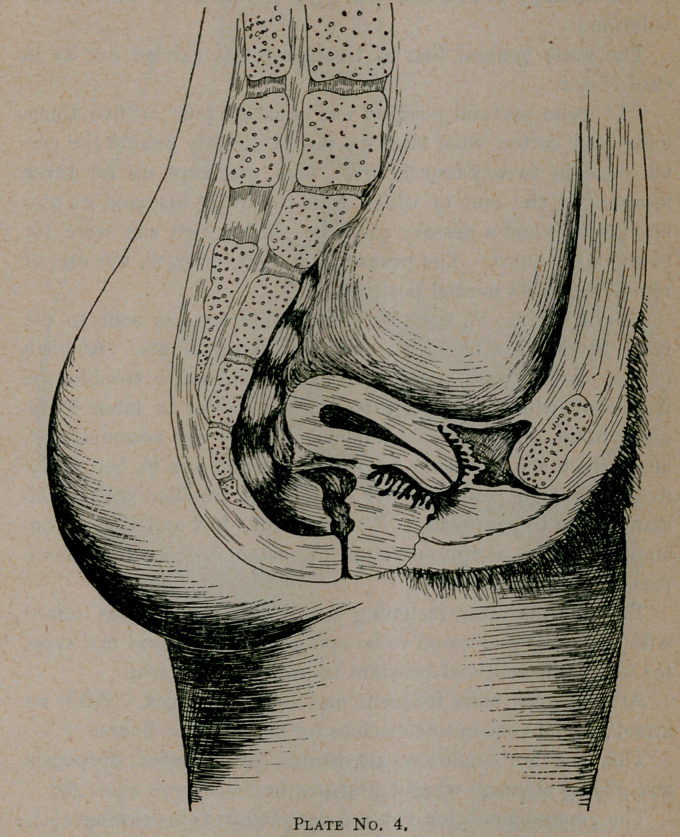


**Plate No. 5. f8:**